# Qualitative research investigating the mental health care service gap in Chinese burn injury patients

**DOI:** 10.1186/s12913-018-3724-3

**Published:** 2018-11-28

**Authors:** Zhengjia Ren, PeiChao Zhang, HongTao Wang, Hongyan Wang

**Affiliations:** 1 Department of Clinical Psychology, The First Affiliated Hospital to Army Medical University (Third Military Medical University), Chongqing, China; 2 Research Center for Modern Psychology, Wuhan University, Wuhan, China; 3 Department of Burn and Cutaneous Surgery, Burn Centre of the People Liberation Army, The First Affiliated Hospital of Air Force Medical University, Xi’an, China; 4 Department of Cadre Ward, Navy General Hospital, Beijing, 100048 China

**Keywords:** Burn injury, Barriers, Mental health care, Sociocultural factors

## Abstract

**Background:**

Psychological disturbances are prevalent in people with burn injuries; however, psychological services are rarely accessiblepost-burn injury in China. The objective of this qualitative study was to explore and conceptualize the obstacles to delivering mental health care in burn injury patients.

**Methods:**

The researchers used a grounded theory research approach to interview sixteen burn injury patients, five nurses, four rehabilitation therapists, five medical doctors, and eight caregivers regarding their experiences with current health care services and barriers.

**Results:**

An explorative model was generated from the data, and the relationships among the categories were identified. People’s beliefs, knowledge, socioeconomic status, cultural understanding of mental health, and social stigma appear to play key roles in the public health approach to post-burn health promotion and post-burn psychosocial interventions.

**Conclusion:**

The model proposed in our research highlights the need to focus on the underlying social, economic, and cultural determinants of mental health care. The underlying social determinants of the mental health care gap that is responsible for the ill-prepared health care must be addressed.

## Background

A significant proportion of burn survivors develop mental disorders, primarily depression and post-traumatic stress disorder (PTSD), at different stages [1, 2]. Studies have shown that approximately one-third of people with burn injuries are affected by depression and PTSD [3, 4]. Psychiatric morbidity among burn survivors increases in direct proportion to the degree of physical and psychosocial disability and the decreases in social and occupational functioning, quality of life, and vitality [5–7]. Accordingly, timely and accurate early screening and diagnosis and appropriate psychosocial intervention for comorbid mental disorders are required not only to increase the quality of life but also to reduce pain and the adverse effects on the course of treatment and increase rehabilitation adherence and efficacy, psychosocial competence, and possibly even prognosis and survival [8, 9]. However, according to previous research, a high proportion of burn survivors with comorbid mental health disorders have not received a diagnosis or treatment for their mental disorders [10]. Many survivors with post-burn psychiatric disorders remain untreated even though effective treatments exist [11]. Although national studies investigating burn epidemiology in China have not been conducted to date, data from several burn centers indicate that the incidence of burn injury is higher in low- and middle-income populations, especially rural populations [12, 13]. Burn survivors in China with a low socioeconomic status are more likely to suffer from physical, social, and psychological impairments [14].

Although many researchers and organizations suggest that good health requires good mental health [15], the integration of mental health awareness into all aspects of health services, health policy, health-system planning and health promotion is largely lacking [16]. The treatment gap for people with mental disorders exceeds 50% in all countries worldwide and approaches astonishingly high rates up to 90% in countries with the fewest resources [17]. According to a World Health Organization (WHO) study, the worldwide treatment gap for major depression and general anxiety disorders (including PTSD) is 56.3 and 57.5%, respectively, and approximately 75% of cases in less-developed countries receive no care at all [18]. Poor and disadvantaged people suffer disproportionately. The resources for mental health care are scarce, and people who are socioeconomically deprived with the highest need for mental health care have the lowest access to mental health services.

Unfortunately, most burn injuries occur among people with a lower socioeconomic status, resulting in objective barriers preventing the recognition of post-burn mental health problems [14]. These barriers include low availability of and accessibility to financial, educational, and material resources [19]; social-cultural factors, such as stereotypes about mental health disorders [20]; and subjective factors, such as stigma and shame [21]. Previous research has shown that shame about mental disorders and even stereotyping mental disorders as weak or bad constrain the use of available resources [20].

The relationship between doctors and patients plays an important role in qualified health-care services. Patient initiated aggression is common among Chinese health professionals, reaching over 10,000 incidents annually, and has become a challenge in maintaining good relationships between health professionals and patients. Tense doctor-patient relationships in the general medical environment generate stress among health professionals, and health professionals have adopted a more passive and self-protective position, which limits patients from receiving more resources and information.

These barriers have resulted in psychosocial-services gap in post-burn care. The current lack of research exploring the treatment gap in burn survivors’ mental health care calls for explorations of the reasons burn survivors who are deeply wounded are left untreated. The purpose of this paper is to explore the perspectives of health care workers, burn survivors, and their caregivers regarding their attitudes, experiences, and beliefs about post-burn mental health services and identify potential gaps that hinder post-burn mental health services in hospitals.

## Methods

### Setting

The data were collected in burn centre of Xijing Hospital, and at a local community center. The patients were recruited from the department of burn and cutaneous surgery, Burn Centre of the People Liberation Army. This department is among the largest burn care centers in China. The community center is a rehabilitation center in the local community that provides rehabilitation services to burn survivors.

### Study design

We followed the systematic procedures recommended by grounded theory to generate new concepts and a theory about the barriers to psychological services for burn injury patients [22]. The participant selection was based on theoretical sampling. Our goal was to select participants who could build our theoretical understanding of the mental health treatment gap from different perspectives. Different perspectives can help increase our understanding of the phenomenon more systematically and comprehensively.

### Data collection and participants

Trained interviewers (ZJ Ren and PC Zhang) conducted in-depth interviews. The data collection and analysis were performed concurrently by ZJ Ren and PC Zhang. Each new participant was selected based on the previous data to ensure new, relevant questions and emerging concepts [22]. Probe questions were used to clarify the participants’ experiences and explore new areas. Interviews were conducted with participants of various ages and levels of education, and variations n other socioeconomic variables, such as different professions, were considered because different professions have different working duties, views and experiences, which may contribute to the service gap. Our research included sixteen burn survivors, five burn surgeons, four rehabilitation therapists, five nurses, and eight caregivers.

Demographics of the patients were presented in Table 1. Of the 16 burn survivors, 11 survivors were male, and five survivors were female. On average, the burn survivors were aged 34.5 years (range 25 to 51). The average total body surface area burned was 36.9% (range 4 to 75%). Ten of these participants had facial disfigurement. Eleven of these participants were injured at work, two participants were injured at home, and three participants were injured outdoors. The time since the injuries occurred ranged from one month to 35 years. Three of these participants had a middle school education, three participants had a high school education, and 10 participants had a university education. Three participants lived in the countryside, nine participants lived in a town, three participants lived in a city, and one participant lived on a farm. Detailed demography data of patients were presented in Table 2.

**Table 1:** Demographics of the patients at the time of first interview

Participants	*N* = 16
Gender	
Men	11
Women	5
Mean age (years)	34.5 (25–51)
Marital status	
Married	10
Divorced	1
Single	5
Education	
University/ College	10
High school	3
Junior middle school	3
Employment	
Working full time	7
Unemployed	7
Self-employed	2
Personal income per month (RMB)	
≥ 5001	1
4001–5000	4
3001–4000	5
2001–3000	3
≤ 2000	2
No stable income	1
Geographical location	
City	3
Town	9
Countryside	3
Farm	1
Average burn area (%TBSA)	36.9% (4–75%)
Location of the burn	
While working	11
Staying at home	2
Outdoors	3

*RMB* Renminbi (Chinese yuan), *TBSA* total body skin area

**Table 2:** Detailed demography data of patients

P	Sex	Education	Marriage	Job	Time of Injured	Place of Injured	Disfigurement	TBSA
P1	Male	U	Married	Self-employed	2 years	Outdoor	No	34
P2	Male	M	Married	Unemployed	1 month	Work	No	26
P3	Female	U	Divorced	Unemployed	16 months	Work	Yes	75
P4	Female	U	Single	Full time	6 years	Work	Yes	15
P5	Male	H	Single	Full time	15 years	Work	No	56
P6	Female	U	Single	Unemployed	7 years	Outdoor	Yes	28
P7	Male	M	Married	Unemployed	5 years	Work	Yes	55
P8	Male	U	Married	Full time	22 months	Outdoor	No	39
P9	Male	H	Single	Unemployed	18 months	Work	Yes	60
P10	Female	U	Married	Full time	3 months	Work	Yes	31
P11	Male	U	Married	Full time	7 months	Work	Yes	4
P12	Male	H	Married	Full time	4 months	Work	Yes	47
P13	Male	U	Single	Unemployed	12 years	Home	No	25
P14	Male	M	Married	Self-employed	10 years	Work	No	39
P15	Male	U	Married	Unemployed	10 months	Work	Yes	34
P16	Female	U	Married	Full time	35 years	Home	Yes	22

*Education U* University/ College, *M* Middle School, *H* High school, *TBSA* total body skin area

The health care professionals included five nurses, four rehabilitation therapists, and five medical doctors. Nine medical professionals were female, five medical professionals were male, and the mean age was 38 years. All health care professionals had extensive working experience in the discipline, and four participants were certified medical doctors. The researcher also interviewed eight caregivers, five of whom were male with a mean age of 47 years. Three caregivers were family members of the patient. Six caregivers had a middle school education, and the remaining two caregivers had a high school education.

The interviews ranged from 30 min to two hours and were audio-recorded. Memos, written notes, and nonverbal information were recorded and used for further analysis.

### Ethical considerations, consent and permissions

This study was approved by the Clinical Ethical Committee of Xijing Hospital, Fourth Military Medical University, Xi’an, China. The potential interviewees were informed of the aims and method of this study in an invitation letter. Prior to participating in the current research, an informed consent form was signed by all the participants (burn survivors, burn surgeons, rehabilitation therapists, nurses, and caregivers) before the interviews started. The interviews were conducted at a hospital and a community center, and the confidentiality and anonymity of the participants were guaranteed by strictly following research procedures and ethical guidelines.

### Data analysis

Audiotapes were transcribed anonymously. The interviews were conducted in Chinese. The interviews were recorded, transcribed verbatim and analyzed inductively based on the analytical process of grounded theory. The information was reviewed and coded [22, 23]. Coding was completed through a series of the following 3 steps: open coding, axial coding, and selective coding [24]. Open coding involves reading and coding the information line-by-line and categorizing the data into codes analytically. Axial coding involves organizing the codes and subcategories through constant comparison and testing the relationships against the data and theory. The theory was derived from the data, and we attempted to identify the parts that fit the data while determining the appropriate theoretical model. After the axial coding, the quote was translated into English by one researcher (HT Wang) and was translated back to Chinese by an English major teacher, and then both of them came to check whether the understanding and translation is consistent. The researcher (HT Wang) is working in local who have experience in the local culture. Selective coding involves forming the theoretical relationships among concepts to generate a theory. During this process, we explored several alternative models and finally selected an explorative model of barriers to mental health services among burn survivors [22]. Here, we also presented the findings to some of the participants, and invited them to provide feedback on the theory. Besides, the researchers also used the self-reflective memos to check the researcher’s own assumptions and biases. In this study, transferability, dependability, and peer checking were used in the analysis process to maintain the trustworthiness of the research.

## Results

**Fig. 1:**
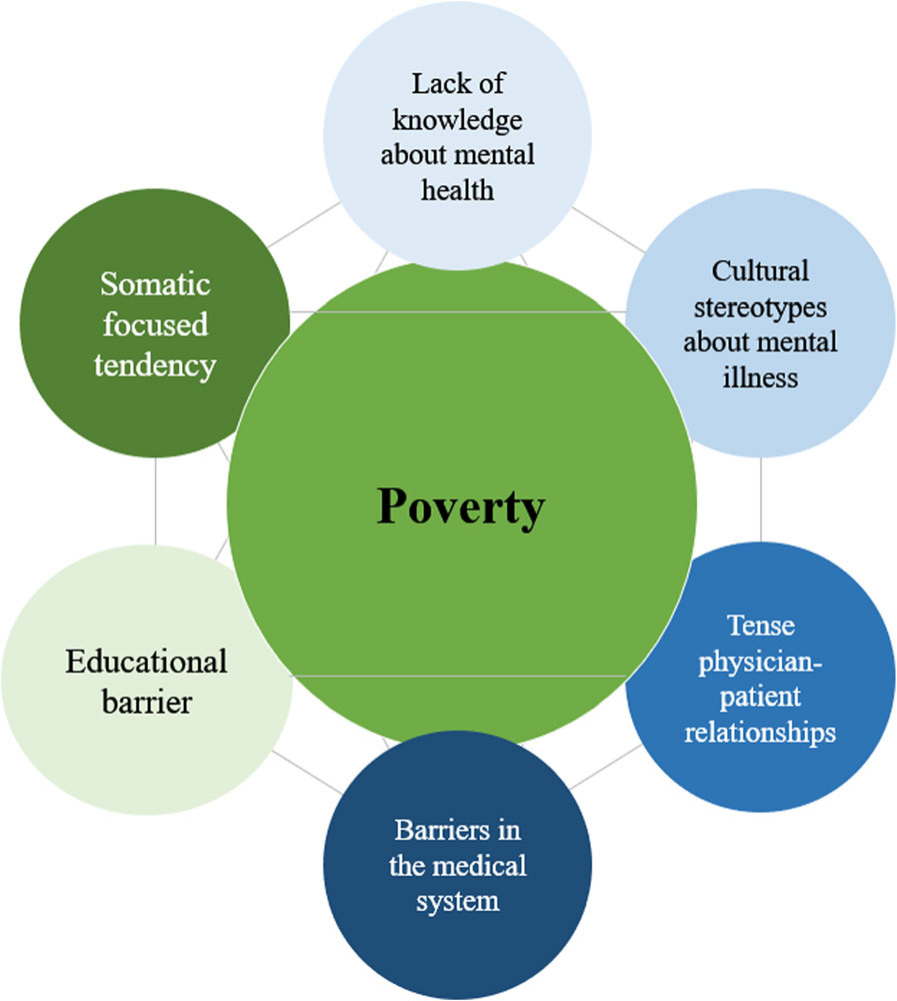
Socioeconomical and Cultural Gaps of Mental Health Services for Burn Survivors in China

Based on our data, we were able to generate an explorative model of gaps in mental health treatment. The basic components of this model (socioeconomical and cultural gaps of mental health services for burn survivors in China) are presented in Fig. 1. We identified the following underlying social, economic, and cultural barriers: lack of knowledge about mental health, barriers in the medical system, tense physician-patient relationships, cultural stereotypes about mental illness, lack of education, somatic focused tendency, and poverty.

### Lack of knowledge about mental health

Lack of knowledge about mental health problems (what, when, where and how such problems occur) was a prominent barrier among all participants. Based on their subjective experiences, most participants had no knowledge about issues relevant to mental health problems, such as the diagnosis of psychological disturbances, the importance of mental health services, and when, where and how to obtain such services.

*One of the patients said, “During one period, I felt that I had no hope. I thought about suicide many times… I was so eager to talk to someone in that moment. I also wanted to talk to a psychologist. But, I do not know where I can find a psychologist.” P2*

This problem was not limited to the burn survivors and caregivers and was also present among the medical professionals. Many of the professionals and caregivers interviewed were not familiar with depression, PTSD, and other psychological disorders. When asked if she knew the symptoms that can follow a traumatic incident, *one nurse said, “They may feel depression.*” However, none of the medical professionals could provide a clear answer regarding the symptoms of depression. Their knowledge about psychological disorders was based on their own common sense. When the surgeons were asked about their knowledge of mental health issues, many shared the sentiment of one surgeon.

*One burn surgeon said, “It is beyond my capability. I know nothing about that.” S1*

*Interestingly, another surgeon said, “I don’t even know where I can find a psychologist.” S2*

*One patient said, “I had asked one doctor to refer me to see psychologists. But, they totally denied me and said they did not know about those resources.”P3*

Altogether, this lack of knowledge among patients, caregivers, and medical professionals limits patients’ access to psychological services.

### Barriers in the medical system

Although mental health services are supposedly a very important part of medical services in hospitals, mental health services for burn survivors are neglected in China. The lack of routine care (screening, diagnosis, and treatment) for psychological problems in hospitals as an objective barrier is evident. The reasons may be due to clinicians being overburdened by their clinical work and their lack of mental health training, which together limit the referral of patients to suitable mental health services. When asked whether they had cooperated with psychological professionals, most clinicians said, *“No, we do not work together with psychiatrists.”*

*Several clinicians said, “I rarely refer patients to psychologists or psychiatrists.”* When the nurses were asked if they provide some psychosocial nursing to burn patients, *one nurse said, “Every day, I need to take care of so many patients, but we do not have very many nurses, and the patients are seriously sick. We cannot handle it all, and we are overburdened.” N1*

*A female patient said, “I see that those nurses are very busy. Everyone in the ward asks for help from them. I want to ask for help because I am also in pain… maybe also frightened… but… they are too busy. They answer me like a robot.” P10*

*One doctor said, “I never do that [refer to mental health services] because I don’t get anything from making referrals to mental health services, so why would I bother to do that?” S1*

Referrals to mental health resources are scarce, and even psychological professionals may not have enough training.

For instance, one clinician said, *“I had transferred one patient to a psychiatrist, and the psychiatrist just encouraged her to think positively. That is all. So, the patient said it doesn’t help.” S5*

The narrative claiming that mental health service providers are ill-equipped to provide services to burn survivors creates a barrier to providing mental health services in the medical system. This barrier was clearly indicated by the patients and caregivers as well as the health service providers involved in this study.

### Tense physician-patient relationships

Medical practice has become a high-risk job in China. Doctors’ legitimate rights and interests are not well protected. Many doctors are under the threat of intimidation and violence, and several doctors have been killed because their patients were not satisfied with their medical services. Several participants shared complaints about this situation.

One nurse said, *“I do not want to trouble myself. If I let the patient see a psychiatrist or psychologist that their caregiver doesn’t like, they may sue me. So, I will not do that, even though I want to at times… I will not do that.”* N4

One doctor said, *“You know, it is beyond my professional duties [to provide mental health services or referrals]. I do not want to do anything that may cause a medical dispute. It is better to avoid trouble.”*S1

Due to tense health care provider-patient relationships, medical professionals assume self-protective positions. These professionals will not risk offering suggestions that may offend the patient.

### Cultural stereotypes about mental illness

Mental illness and the people who suffer from mental illness are viewed negatively in Chinese culture. Individuals with mental illness are considered “mad”, “crazy”, “weak-willed” or even “psychotic”. These stereotypes were prevalent among our participants.

One patient said*, “If I went to see the psychologist or psychiatrist, other people would think I am not only physically distorted [by my burn injury] but also psychologically distorted.” P9*

One nurse said, *“We cannot let [patients] know that they need to see a psychologist or psychiatrist. They cannot accept that. They may think that I think he or she is mentally ill.” N3*

One rehabilitation therapist said, *“I am not sure they [the patients] would like to see mental health professionals. They may see them secretly. I do not [refer them] because I do not want to hurt their self-esteem. If I told them to see mental health professionals, they might feel inferior.” R2*

One doctor said, *“I won’t refer my patient to psychological professionals, even though I think it is necessary. And, you know, if I tell someone to see a psychologist or psychiatrist, it sounds weird, like I am calling them names. It feels like [I] think someone has psychotic problems [is crazy]. I rarely suggest that in my career.” S3*

During the interview, the patients felt very frightened and asked the interviewer questions, such as *“Do you think I have psychological problems?”* The patients were afraid to be labeled as mentally ill.

One patient said, *“If I know I have psychological problems, I will be very depressed.” P4*

Another patient said, *“If I have psychological problems, I hope my doctor does not tell me. Maybe I do, but my doctor wants to help me, and he wants to protect me, so he did not tell me. I think it is good for me to not know about that.” P9*

Psychological problems are usually perceived as a “weakness” and “incompetence.”

One caregiver said, “*My son is very strong and has a strong will. He bears all the pain by himself. He never cries. He does not need mental health help*.”*C2*

Stigma about mental disorders constrains the availability and utilization of mental health services among both patients and medical professionals.

### Educational barrier

Most participants had limited education and training about the importance of mental health services.

One patient with an undergraduate degree said*, “When I was in university, we had a course called “Introduction to Psychology.” It was very simple. I totally forgot and have no impression about that course.”P11*

The “educational barrier” reflects the lack of basic psychological knowledge and skills in the general population and the insufficient psychological training of medical professionals.

### Somatic focused tendency

Eastern culture emphasizes the monism of mind and body. The body is the container of the psyche, and most participants pay more attention to their bodies, including their appearance, body functions, and sensations, than to their psychological problems.

One caregiver said*, “I believe my kid is brave. He is much braver than me. Throughout the whole rehabilitation process and hundreds of operations… he never complained. I believe if his function and appearance recover, he will never have any psychological problems.” C1*

A female burn survivor said*, “My ear was injured [burned] when I was five years old. I always felt inferior compared to other people. It influenced my career and mate selection…My husband, he never asked what is wrong with my injury…I never discussed it with him, and I never told anyone about that [crying]…I had plastic surgery seven times, but it is still not good enough in the way I want…I will do the surgery again. If the surgeries are successful, I will not have any psychological problems.” P16*

Many patients suffered from post-traumatic stress but did not pay attention to their mental health. The patients believed that if they could repair their disfigurement, they would experience no psychological disturbances. In their ideology, the body is the mind, and the mind is the body.

### Poverty

Most burn survivors have a low socioeconomic status. The high costs of treatment and rehabilitation aggravate their socioeconomic situation, leading to more severe poverty. Most patients spend all their money on basic physical treatment, leaving no money for mental health treatment or physical rehabilitation.

When asked about the patients’ and their families’ economic plight, one of the surgeons who has worked at the hospital for ten years said*, “Most burned people live in the lowest social class. After the injury, they have no money for basic physical treatment, much less (money) for psychological rehabilitation.” S4*

The burden of burn injuries overwhelms poor families.

One caregiver said*, “We were farmers. After the incident, we came to work in the city for almost four years [to earn the operation fee]. We worked day and night, but we can only afford this times’ operations for my son [crying]. There is still a lot that the surgeons need to do…We do not want to divert money from expensive [surgeries].” C8*

These injuries are partially triggered by poverty, and the cost of treatment worsens poverty. Patients cannot afford a basic living, much less mental health services.

## Discussion

The World Health Organization (WHO) has identified psychological rehabilitation as an essential and integral component of general health. The gap in mental health services among burn survivors in China prevails [25], and health professionals need to identify the underling barriers and translate research results into practice to meet the needs of burn survivors [26]. The present theoretical model found the overlapped barriers which prevented the burn survivors get the best psychological rehabilitation services, and the theoretical model also discovered that the underlying economic barrier is the decisive obstacle of mental health services gap.

Research in the field of cultural psychopathy has found that a tendency exists for Chinese individuals to emphasize somatic interpretations of psychological disturbances [27]. Due to the somatic-focused culture, most physicians and caregivers focus on the physical needs of burn patients while failing to identify the psychological needs. The perceived stigma associated with mental illness plays an important role in the help-seeking behaviors of patients [28]. However, clinicians’ stereotypes related to mental illness may also partially prevent medical professionals from referring patients to psychological professionals.

Chinese culture emphasizes harmony in interpersonal relationships and the suitable regulation of relationships [29]. The principal of regulation of relationships refers to following the reciprocity principal in hierarchically-structured social relationships, thereby allowing individuals to strive for desirable resources [30]. Violence toward medical professionals and the increase in medical lawsuits violates the reciprocal principal of *relationships,* and disregarding this reciprocity damages helping behavior by leading medical professional to assume a more self-protective and self-defensive position [31, 32]. These antagonistic relationships not only lower the emotional support provided to the patient but also lead to more conservative professional behaviors.

Various studies focused on assessing the knowledge of healthy behaviors among patients have implied that people who are more aware and have more knowledge about their problems will engage in healthy activities [33]. The lack of knowledge about mental health problems among caregivers, patients, and even medical professionals limits patients’ access to mental health services. These treatment gaps highlight that mental health care and knowledge of psychopathology need to be further developed. Current research suggests that more training in psychopathology at the undergraduate level, continuing education at the burn injury ward, and vocational training for medical professionals are warranted in China [20, 25].

Recent guidelines for burn rehabilitation emphasize the multidisciplinary teamwork model in burn care units in China, which should include all stakeholders, including patients, surgeons, nurses, physical and occupational therapists, rehabilitation nurses, psychiatrists, psychotherapists, nutritionists, social workers, and families, to maximize the rehabilitation of patients [25]. The reformation of the burn injury care system is necessary. However, we cannot deny the fundamental impact of poverty on the lives of burn survivors and their families. Poverty is associated with an increased risk of burn injuries, and the expense of post-burn injury worsens living situations [34, 35]. Out-of-pocket health payments for severe conditions remain a tremendous burden on people with a low socioeconomic status, and financial protection from the New Cooperative Medical Scheme is limited in its effectiveness, particularly for the poorest populations, due to low levels of reimbursement, lack of funding, improper management, poor access, and complicated system of provision [36–38]. Our research further verifies that patients and their family members are unable to meet their basic needs because of the high expense of medical services related to burn treatment and rehabilitation. The financial hardship limits their access to mental health services, their education about mental health, and their ability to build and sustain social relationships. The financial hardship further increases their cultural stigma and feeling of shame.

The model proposed in our research highlights the need to focus on the underlying social, economic, and cultural barriers to mental health care. The barriers identified in our research largely determine the combined actions of burn survivors who are prevented from obtaining mental health care, and therefore, the focus of health promotion and policy should be on addressing these barriers that create the mental health services gap.

## Conclusions

Our research identifies the underlying social, economic, and cultural barriers that contribute to poorly implemented mental health care among burned patients. The promotion of mental health services in Chinese hospitals is slowly evolving, but based on our model, one of the fundamental barriers to effective mental health promotion is poverty. Further health policy reform is needed to provide more support to households trapped in poverty due to out-of-pocket health payments. Personal stigma and physicians’ fear of referrals for mental health are obvious and need to be addressed through education. However, medical professionals are well placed to promote mental health services both at the individual level and more broadly.

Medical professionals working in burn injury departments lack knowledge, awareness, and education about mental illness. This gap in China must be urgently addressed partially by modifying the medical curriculum to include relevant psychological material and encouraging medical professionals to undertake further training in this field. Cultural challenges should be addressed through cultural sensitivity services in future health promotions and health policy planning. The tense health care provider-patient relationships need to be solved by building mutual trust and cultivating medical professionals′ communicating ability. Future research related to health promotion and health policy should aim to further explore these gaps and evaluate the effectiveness of interventions.

The treatment gap in post-burn mental disorders is shaped by various social, economic, cultural, and medical environments that are heavily associated with poverty in China.

## Abbreviations

C: Caregiver
N: Nurse
P: Patient
PTSD: Post-traumatic stress disorder
R: Rehabilitation therapist
S: Burn Surgeon
WHO: World Health Organization
